# Chloroplast genome of *Tillandsia landbeckii* Phil. (Bromeliaceae) a species adapted to the hyper-arid conditions of the Atacama and Peruvian desert

**DOI:** 10.1080/23802359.2021.1997122

**Published:** 2021-11-10

**Authors:** Julio César Chávez-Galarza, Steffany Cardenas-Ninasivincha, Roberto Contreras, Rubén Ferro-Mauricio, Wilson Huanca-Mamani

**Affiliations:** a Laboratorio de Investigación Tecnológica en Cambio Climático, Instituto Nacional de Innovación Agraria. Av, Lima, Perú; b Departamento Académico de Ciencias Básicas y Afines, Universidad Nacional de Barranca. Av. Toribio de Luzuriaga 376, Barranca, Perú; c Laboratorio de Biología Molecular de Plantas, Facultad de Ciencias Agronómicas, Universidad de Tarapacá, Arica, Chile; d Centro Regional de Investigación y Desarrollo Sustentable de Atacama (CRIDESAT), Universidad de Atacama, Copiapó, Chile

**Keywords:** Chloroplast genome, *Tillandsia landbeckii*

## Abstract

*Tillandsia landbeckii* Phil. is a vulnerable species belonging to the terrestrial Bromeliaceae family; it is highly adapted to extremely hyper-arid conditions of the Atacama Desert and Peruvian deserts. In this study, we sequenced, assembled, and annotated its chloroplast genome. *T. landbeckii* chloroplast genome is 159,131 bp in length, containing a large single-copy region of 87,164 bp, a small single-copy region of 18,521 bp, and a pair of inverted repeat regions of 26,723 bp. The GC content of the chloroplast genome is 37.33%. It encodes a total of 132 genes, including 86 protein-coding genes, 38 tRNA genes and 8 rRNA genes. The phylogenetic tree indicates that *T. landbeckii* is placed within the Bromeliaceae family and has a close relationship with *T. marconae* with 100% support.

*Tillandsia landbeckii* is a perennial herbaceous species highly adapted to the Atacama Desert and South-Peruvian deserted areas' extremely hyper-arid conditions. It is distributed naturally from Lima Department, central of Peru, to Coquimbo region, north of Chile (Rundel and Dillon 1998; Pinto 2005). Due to the absence of functional roots, this species grows unattached to the sandy soil surface and absorbs the regular fog humidity through specialized leaf trichomes to survive in the arid coastal region of the Atacama and Peruvian desert (Rundel et al. 1997; Pinto et al. 2006). *T. landbeckii* occupies a unique ecological niche, which makes it particularly vulnerable to the effects of climate change and habitat destruction. Because of its extreme specialization and dependence on fog humidity, this species is considered as a‘vulnerable species’ in both countries (Zizka et al. 2009; Whaley et al. 2019; MMA 2021). So far, the chloroplast genome of *T. landbeckii* is not available. In this study, we sequenced and characterized the chloroplast genome of *T. landbeckii* to serve as a genetic resource for future studies on the taxonomy and to get a better understanding of phylogenetic relationships in the *Tillandsia* genus.

Sample of *T. landbeckii* was collected from Pampa Camarones, Arica, Chile (18° 52′ 31.40′ S; 70° 6′ 32.30′ W) and a specimen of *T. landbeckii* is conserved in the herbarium of Universidad de Concepción, Chile (http://www2.udec.cl/∼herbconc/, Dra. Alicia Marticorena, amartic@udec.cl) under the voucher CONC-184217. Total genomic DNA was extracted from fresh leaves using the DNeasy Plant Mini Kit (Qiagen, Hilden, Germany) following the manufacturer's instructions. DNA library was sequenced, and 150 pb paired-end reads were generated on an Illumina NovaSeq PE150 platform. After base quality control using FASTQC v01 (Andrews 2010), the remaining high-quality reads were used to assemble the chloroplast genome by SPAdes (Bankevich et al. 2012) and NOVOPlasty v01 (Dierckxsens et al. 2017), using *T. usneoides* chloroplast genome as a reference (KY293680.1). The chloroplast genome annotation was performed by GeSeq (Tillich et al. 2017) (Figure 1).

**Figure 1.**
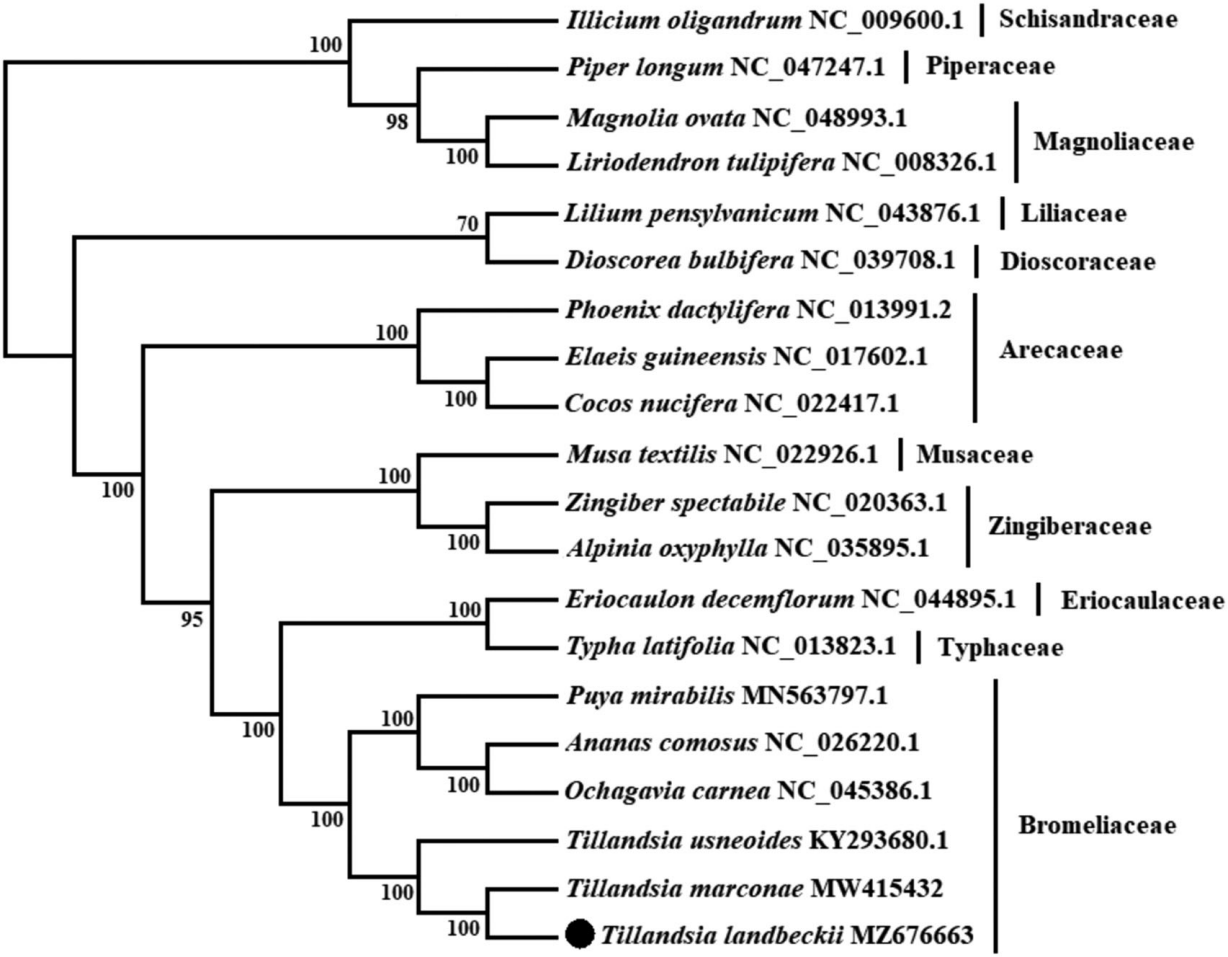
Maximum likelihood (ML) tree based on chloroplast genome sequences of 20 species. Values along branches correspond to ML bootstrap percentages.

The chloroplast genome of *T. landbeckii* (GenBank accession number: MZ676663) is 159,131 bp in length with 37.33% GC content, displaying a quadripartite structure that contains a pair of inverted repeats (IR) regions (26,723 bp, GC content 42.68%), separated by a large single-copy (LSC) region (87,164 bp, GC content 35.31%) and a small single-copy (SSC) region (18,521 bp, GC content 31.46%). A total of 132 genes were predicted, including 38 tRNA genes, 8 rRNA genes, and 86 protein-coding genes.

A phylogenetic analysis was performed using chloroplast genomes from 20 species and with *Magnolia ovata*, *Liriodendron tulipifera*, *Illicium oligandrum,* and *Piper longum* as outgroup. The genomes were aligned using MAFFT version 7.475 software (Katoh and Standley 2013), involving the concatenation of 74 orthologue protein-coding genes. Five species from Bromeliaceae, three from Arecaceae, two from Zingiberaceae, and one each from Typhaceae, Eriocaulaceae, Musaceae, Liliaceae, and Dioscoreaceae corresponding to Liliopsida class were included in the analysis. The phylogenetic analysis was conducted based on maximum-likelihood (ML) analysis using IQ-Tree software (Nguyen et al. 2015) under GTR + I + G nucleotide substitution model, which was selected by Jmodeltest 2 (Darriba et al. 2012). The ML tree was inferred by bootstrapping with 1000 replicates. The phylogenetic tree showed that *T. landbeckii* is placed under the Bromeliaceae family and has a close relationship with *T. marconae* with 100% support. This chloroplast genome will contribute to conservation, phylogenetic and evolutionary studies in desert plant species.

## Data Availability

The data that supports this study is openly available in GenBank of NCBI (https://www.ncbi.nlm.nih.gov) under the accession number MZ676663. The associated Bio project, Biosample, and SRA numbers are PRJNA742530, SAMN19959867, and SRR15015612, respectively.
